# Dissolved black carbon is not likely a significant refractory organic carbon pool in rivers and oceans

**DOI:** 10.1038/s41467-020-18808-8

**Published:** 2020-10-07

**Authors:** Yuanzhi Qi, Wenjing Fu, Jiwei Tian, Chunle Luo, Sen Shan, Shuwen Sun, Peng Ren, Hongmei Zhang, Jiwen Liu, Xiaohua Zhang, Xuchen Wang

**Affiliations:** 1 Key Laboratory of Marine Chemistry Theory and Technology, Ministry of Education/ Center for Frontier Science of Deep Ocean and Earth System, Ocean University of China, Qingdao, China; 2 Key Laboratory of Physical Oceanography, Ocean University of China, Qingdao, China; 3 Center for Isotope Geochemistry and Geochronology, Qingdao National Laboratory for Marine Science and Technology, Qingdao, China; 4 Key Laboratory of Marine Genetics and Breeding, College of Marine Life Sciences, Ocean University of China, Qingdao, China

**Keywords:** Biogeochemistry, Ocean sciences

## Abstract

Rivers are the major carriers of dissolved black carbon (DBC) from land to ocean; the sources of DBC during its continuous transformation and cycling in the ocean, however, are not well characterized. Here, we present new carbon isotope data for DBC in four large and two small mountainous rivers, the Yangtze and Yellow river estuaries, the East China Sea and the North Pacific Ocean. We found that the carbon isotope signatures of DBC are relatively homogeneous, and the DBC ^14^C ages in rivers are predominantly young and increase during continuous transport and cycling in the ocean. The results of charcoal leaching experiments indicate that DBC is released from charcoal and degraded by bacteria. Our study suggests that riverine DBC is labile and respired during transport and mixing into the ocean and that residual DBC is cycled and aged on the same time scales as bulk DOC in the ocean.

## Abstract (web summary)

Black carbon is a recalcitrant byproduct of biomass burning that ultimately accumulates in oceanic sinks. Here the authors assessed the sources and cycling of dissolved black carbon in rivers and oceans, finding that oceanic pools are cycled and aged on the same time scales as dissolved organic carbon.

## Introduction

Black carbon (BC), a common residue of incomplete combustion of biomass and fossil fuel, is widely dispersed in soils and marine sediments^1,2^. Defined as a group of condensed byproduct chemicals, BC is thought to be chemically stable and hardly biodegradable^3,4^, thus representing a refractory fraction of organic carbon (OC), and its role in the global carbon cycle and its environmental impacts have received considerable attention^2,5–8^. In recent years, several studies have reported that dissolved BC (DBC) could represent a significant fraction of BC mobilized and transported by rivers into the ocean^9–12^. World rivers are estimated to carry 27 Tg yr^−1^ of DBC, which accounts for approximately 10% of the global flux of dissolved OC (DOC) via rivers^12^. The amount of river transported DBC is also equipollent to the particulate BC (PBC) transported by rivers worldwide (17–37 Tg yr^−1^)^6^. Together, the annual transport of DBC and PBC by rivers could account for 8–60% of the annual production of BC, thus representing a dominant source of BC input into the oceans. Globally, it is estimated that the production of BC is dominated by biomass burning (114–383 Tg yr^−1^), which is one to two orders of magnitude higher than the amount of BC produced from fossil fuel combustion (2–29 Tg yr^−1^)^6^. In their recent study, Jones et al.^13^ estimated that 60 Pg of BC (or pyrogenic carbon) has been produced cumulatively since 1750 from landscape fires, accounting for 33–40% of the global biomass carbon lost through land use and deforestation in the same time period^13^. This carbon-rich charcoal can be stored in soils and oceans over very long time periods^2,14^, thus representing a significant, but overlooked, sink for atmospheric CO_2_^13^. Although the size of DBC exported by rivers is still a relatively small fraction of the DOC pool in the global carbon cycle and budget, its geochemical importance is thought to be significant due largely to its stable chemical structure, resistance to microbial degradation^15,16^, and long residence time as a major sink of atmospheric CO_2_ in the global carbon cycle^10,11,17,18^

The sources and fate of DBC in the natural aquatic environment have not been well constrained due mainly to the lack of a well-recognized and consensus standard separation and the quantification method^19,20^. The chemothermal oxidation (CTO) method has been used as a popular isolation technique to determine BC in environmental samples^21–23^. This method determines mainly highly condensed aromatic compounds of BC^20^. In recent years, benzene polycarboxylic acids (BPCAs) have also been used as molecular proxies to quantify DBC, mainly polycyclic aromatic compounds, in both rivers and oceans^9–11,24,25^. Despite the different techniques used, the abundances of DBC reported by the two methods show high similarity in both river and oceanic waters^9,17,24–26^.

Radiocarbon (^14^C) measurement has been used as a powerful tool to distinguish the sources of DBC produced from biomass burning and fossil fuel combustion because these two sources have distinct ive ^14^C ages (modern vs. ancient)^2,9–11^. The DBC isolated from rivers has been found to have relatively modern ^14^C ages^9,26^, while the ^14^C ages of DBC in the ocean are extremely old (>20,000 years)^10,11^. These studies raised some important questions that have not been answered. Why are the ^14^C ages of DBC so different in rivers and oceans if world rivers are the major pathways of transporting and exporting DBC from land to ocean? Could the extremely aged ^14^C DBC in the ocean suggest that DBC has been cycled for a much longer time than the bulk DOC in the ocean? In a recent study, Wagner et al.^24^ measured the stable carbon isotopes of DBC in rivers and oceans. They found large differences in the δ^13^C compositions of DBC that were more depleted (by −6‰) in rivers than in the ocean. They concluded that oceanic DBC is unlikely to be sourced from riverine DBC, thus making the oceanic DBC story even more enigmatic. If the majority of DBC in the ocean is not from river inputs, can we determine another possible major source that contributes DBC to the ocean? Our current understanding of the sources and cycling of DBC in the ocean is based on a few studies that seem contradictory rather than consistent.

Here, we report new carbon isotope (Δ^14^C and δ^13^C) results for DBC isolated by solid phase extraction (SPE) from four major rivers in China (Yangtze, Yellow, Pearl, and Heilongjiang rivers) and two small mountainous rivers in Taiwan (Dadu and Tamsui), as well as DBC isolated from the Yangtze and Yellow river estuaries, the East China Sea (ECS) and the Mariana Trench site in the western North Pacific (NP). In addition, long-term (~1700 days) locust tree wood charcoal leaching experiments were conducted to determine the dissolution of DBC from charcoal in river water with and without bacteria and to assess the possible biodegradation of DBC. These data provide new insight for our understanding of the source, biodegradability, and cycling time scales of DBC in rivers and oceans.

## Results

### Concentrations of DOC and DBC in rivers and oceans

**Fig. 1:**
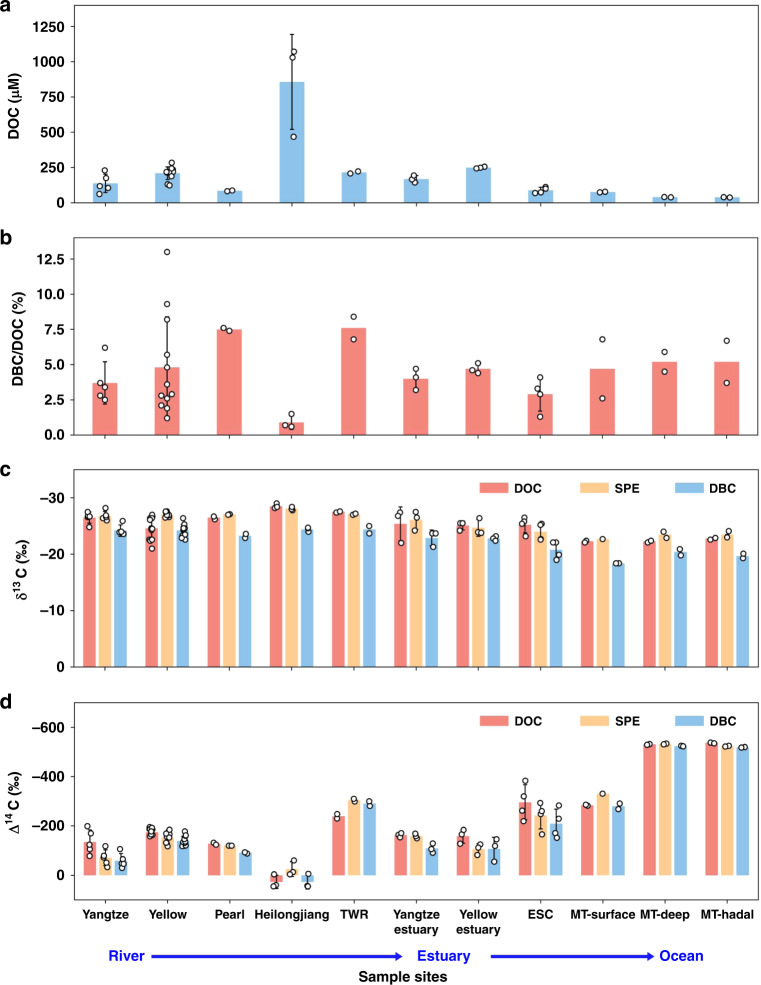
Distribution of the concentrations and isotopic compositions. **a** The concentrations of DOC, **b** DBC/DOC ratios, **c** the δ^13^C values, and **d** the Δ^14^C values of DOC, SPE-DOC, and DBC in the Yangtze (*n* = 5), Yellow (*n* = 12), Heilongjiang (*n* = 3), Pearl (*n* = 2), and Taiwan rivers (TWR, *n* = 2), the Yangtze River Estuary (*n* = 3), the Yellow River Estuary (*n* = 3), the East China Sea (ECS) (*n* = 4), and the Mariana Trench (MT) site in the North Pacific (NP) (*n* = 2 MT-surface, *n* = 2 MT-deep, *n* = 2 MT-hadal) (DOC dissolved organic carbon, SPE-DOC solid phase extracted dissolved organic carbon, DBC dissolved black carbon). The bar charts are the means and the error bars are the calculated standard deviations (SD) of the data group (for *n* ≥ 3). The black circles are corresponding data points.

Sampling locations are summarized in Supplementary Table 1, and the concentrations and isotope data are listed in Supplementary Table 2. The concentrations of DOC in the rivers showed large variations, ranging from 62 to 1072 μM. On average, DOC concentrations were comparable in the Yangtze (138 ± 65 μM), Yellow (210 ± 45 μM), Pearl (84 ± 4 μM), and Taiwan rivers (216 ± 11 μM) but much higher in the Heilongjiang River (857 ± 337 μM) (Fig. 1a). For the monthly samples in the lower Yellow River, DOC concentrations were lower in May and June (123–131 μM) during the high flow period but were quite consistent in the other months of 2015. DOC concentrations were higher in the Yellow River Estuary (250 ± 4 μM) than in the Yangtze River Estuary (168 ± 25 μM) and decreased gradually from the Yangtze River Estuary to the ECS (89 ± 21 μM). At the Mariana Trench site in the western NP, DOC concentrations decreased from 76 ± 2 μM in the upper 100 m to 39 ± 1 μM at great depths (8000–10,000 m) (Fig. 1a).

The SPE extraction efficiencies of DOC also showed great variations from river to ocean waters, ranging from 40 to 76% (Supplementary Table 2). Overall, the SPE extraction efficiencies of DOC were higher for river water (61 ± 6%) than for estuarine and coastal waters (51 ± 11%) and open ocean waters (47 ± 4%). These results are consistent with the values reported in recent studies using the same SPE method^9,24^. DBC contents measured in the SPE-DOC also showed variations in the rivers, and the average DBC/DOC ratios were 3.7 ± 1.5% in the Yangtze River, 4.8 ± 3.6% in the Yellow River, 7.5 ± 0.1% in the Pearl River, 0.9 ± 0.5% in the Heilongjiang River, and 7.6 ± 1.1% in the Taiwan rivers. The Heilongjiang River had the highest DOC but the lowest DBC/DOC ratio (0.9 ± 0.5%). For the monthly Yellow River samples, it appeared that the DBC/DOC ratios were relatively lower (1.2–2.6%) during winter months (January, November, and December) and high flow months (1.9–3.6%, May–July) than during the other months (Supplementary Table 2). The average DBC/DOC ratios were 4.0 ± 0.8% in the Yangtze River Estuary, 4.7 ± 0.4% in the Yellow River Estuary, 2.9 ± 1.2% in the ECS, and 2.6–6.8% in the deep water of the Mariana Trench site in the NP (Fig. 1b).

### Isotopic signatures of DOC, SPE-DOC, and DBC in rivers and oceans

The DOC δ^13^C values showed a wide range from −21.0 to −29.0‰ in the rivers. On average, DOC in the Heilongjiang River had a more depleted δ^13^C value (−28.5 ± 0.4‰) than DOC in the Taiwan (−27.5 ± 0.2‰), Pearl (−26.5 ± 0.4‰), Yangtze (−26.5 ± 1.0‰), and Yellow (−24.6 ± 2.0‰) rivers (Fig. 1c). The Yellow River monthly DOC δ^13^C values also showed large variation, ranging from −21.0 to −27.0‰. The DOC δ^13^C values were on average −25.4 ± 3.0‰ in the Yangtze River Estuary, −25.1 ± 0.8‰ in the Yellow River Estuary, −25.2 ± 1.4‰ in the ECS, and −22.1 to −23.1‰ at the Mariana Trench site in the NP. In contrast, the δ^13^C values of SPE-DOC in each river were mainly comparable with the bulk DOC δ^13^C values for most samples and were also quite constant in each river, especially for the Yellow River monthly samples. The average δ^13^C values of SPE-DOC were −26.8 ± 0.8‰ in the Yangtze River, −27.8 ± 0.4‰ in the Yellow River, −27.0 ± 0.1‰ in the Pearl River, −28.1 ± 0.3‰ in the Heilongjiang River, and −27.1 ± 0.1‰ in the Taiwan rivers (Fig. 1c). The δ^13^C values of SPE-DOC were −26.1 ± 1.7‰ and −24.7 ± 1.5‰ in the Yangtze River and Yellow River estuaries, respectively, −24.0 ± 1.5‰ in the ECS, and −22.7‰ at the surface (50–100 m) and −23.5 ± 0.8‰ in the deep depths (3000–10,000 m) at the Mariana Trench site. These samples showed no significant differences when compared with bulk DOC δ^13^C values. The δ^13^C values of DBC ranged from −22.6‰ to −26.4‰ in the rivers and −21.3 to −23.7‰ in the estuaries, −20.8 ± 1.6‰ in the ECS and −18.4‰ to −20.9‰ in the Mariana Trench site, which are considerably higher than the δ^13^C values of both DOC and SPE-DOC in the rivers and oceans. As plotted in Fig. 1c, the average DBC δ^13^C value was enriched by 3–4‰ compared to those of DOC and SPE-DOC, which was the most notable systematic change in DBC δ^13^C signatures.

Large variations were found in Δ^14^C values among the samples. The DOC Δ^14^C values ranged from 45 to −248‰ in the rivers, −154 to −184‰ in the estuaries, −219 to −383‰ in the ECS, and −282 to −538‰ at the Mariana Trench site in the NP. The corresponding ^14^C ages spanned from modern to 6180 years before present (YBP). On average, the Δ^14^C values of DOC were −135 ± 50‰ in the Yangtze River, −175 ± 15‰ in the Yellow River, −130 ± 5‰ in the Pearl River and −240 ± 10‰ in the Taiwan rivers. The monthly DOC Δ^14^C values in the Yellow River were slightly higher in the high flow season (May–July) but were mostly consistent across months in 2015. The DOC in the Heilongjiang River, however, had a modern Δ^14^C value of 27 ± 27‰ (Fig. 1d). The average DOC Δ^14^C values were −160 ± 10‰ and −159 ± 29‰ in the Yangtze River and Yellow River estuaries, −295 ± 70‰ in the ECS and −284 ± 2‰, −531 ± 2‰, and −537 ± 2‰ in the surface, deep and hadal depths at the Mariana Trench site (Fig. 1d). The ^14^C age of DOC in the depths (8000–10,000 m) of the Mariana Trench site was ~6100 years. In contrast, the Δ^14^C values of SPE-DOC in the Yangtze (−70 ± 30‰), Yellow (−150 ± 20‰), and Pearl (−120 ± 1‰) rivers were slightly higher (or younger) than those of DOC but slightly lower (older) than those of DOC in the Heilongjiang (−25 ± 30‰) and Taiwan rivers (−305 ± 10‰). The SPE-DOC Δ^14^C had values similar to those of DOC in the Yangtze River Estuary (−160 ± 10‰), were higher in the Yellow River Estuary (−106 ± 22‰) and the ECS (−240 ± 50‰) and were comparable to those of DOC in the Mariana Trench of the NP (−331, −533, and −524‰) (Fig. 1d).

The Δ^14^C values of DBC also showed notable variations (Fig. 1d). The average DBC Δ^14^C values were −60 ± 30‰ in the Yangtze River, −140 ± 20‰ in the Yellow River and −91 ± 3‰ in the Pearl River, which were 30–65‰ higher (~200–500 years younger) than those of DOC and slightly higher than those of SPE-DOC (Fig. 1d). For the Yellow River, the DBC Δ^14^C values in each month were all higher than the Δ^14^C values of DOC (by 6–77‰). The DBC Δ^14^C value was modern (27 ± 29‰), the same as that of DOC in the Heilongjiang River, and −290 ± 10‰ (50‰ lower or 350 years older) relative to those of DOC in the two small mountainous rivers in Taiwan. The DBC Δ^14^C values in the Yangtze River Estuary (−110 ± 20‰), Yellow River Estuary (−107 ± 47‰), and the ECS (−210 ± 60‰) were also significantly higher (by 50‰ to 85‰) or younger (by ~350–660 years) than those of DOC. At the Mariana Trench site in the NP, the DBC Δ^14^C values on average were −80 ± 20‰ in the surface water (50–100 m), −524 ± 1‰ in the deep (3000–6000 m) and −519 ± 1‰ in the hadal (8000–10,000 m) waters. The values showed almost no differences from those of DOC (Fig. 1d).

### Leaching and biodegradation of DBC

**Fig. 2:**
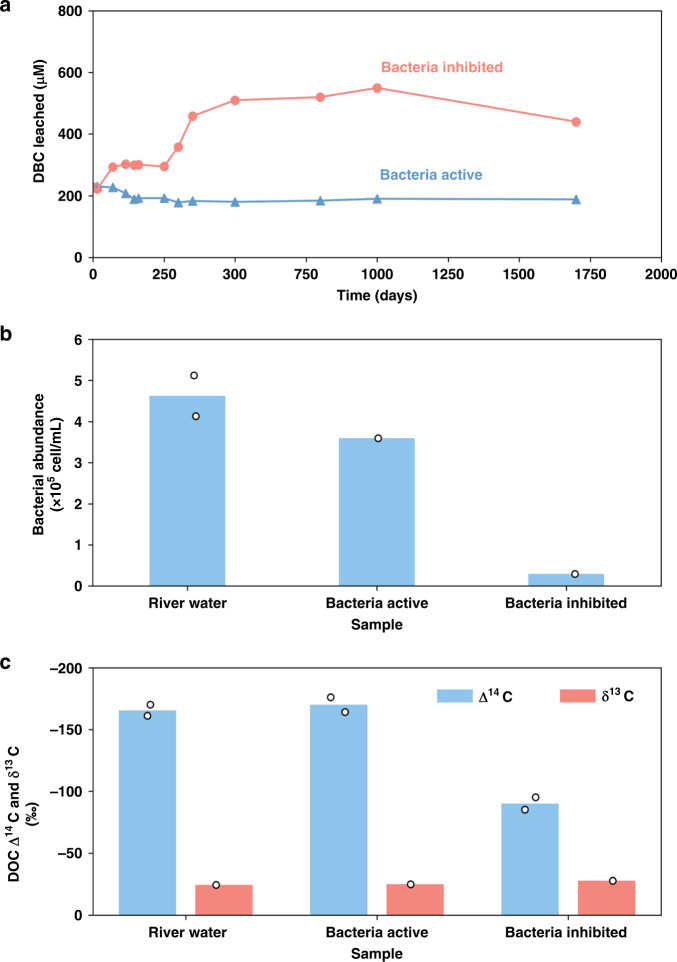
Characteristics of DBC in the leaching experiments. **a** Long-term time incubation showing the leaching of DBC (dissolved black carbon), measured as DOC (dissolved organic carbon) concentrations from charcoal in both bacteria-active and bacteria-inhibited river waters during the 1700-day incubation period; **b** bacterial abundance, and **c** Δ^14^C and δ^13^C values of DOC measured in the river water and bacteria-active and bacteria-inhibited river waters at the end of the experiments. The bar charts are the average values of the duplicate measurements (true value for single point measurement). The black circles are corresponding data points.

The results of the long-term charcoal leaching experiments are plotted in Fig. 2. It can be seen that significant DBC was released and dissolved in DOC from the charcoal in the bacteria-inhibited case (Fig. 2a). It appeared that two steps were involved in the charcoal release processes: the first release took place in the first 100 days, with an increase of approximately 70 μM DOC, and the second release took place after 250 days, with 550 μM DOC measured at 1000 days (~322 μM DOC increase from the initial 228 μM). The locust tree wood charcoal was C-rich, and its C% was measured at 80%. We calculated that 2.4% of the charcoal C was dissolved in the DOC on day 1000. At the end of the experiment, the DOC concentration was measured for 440 μM in the bacteria-inhibited case, a 20% decrease from the highest level of 550 μM. In comparison, for the significant difference in the bacteria-active leaching case, the DOC concentration actually decreased by 17% after 150 days from the initial 228 to 188 μM and then remained unchanged to the end of the experiment (Fig. 2a). No DBC accumulation was observed in the bacteria-active water.

The bacterial abundance measured in the bacteria-active water was much higher (3.58 × 10^5^ cell mL^−1^) than that in the bacteria-inhibited water (0.28 × 10^5^ cell mL^−1^) at the end of the experiments (Fig. 2b). The bacterial abundance was 4.63 × 10^5^ cell mL^−1^ in the Yellow River water measured as the reference (Fig. 2b). For DOC isotopic values, the Δ^14^C and δ^13^C values were measured at −170 and −25.1‰ in the bacteria-active water and −90 and −27.8‰ in the bacteria-inhibited water at the end of the experiments, and these values were compared with the initial DOC Δ^14^C and δ^13^C values of −166 and −24.6‰ of the Yellow River water, respectively (Fig. 2c).

## Discussion

**Fig. 3:**
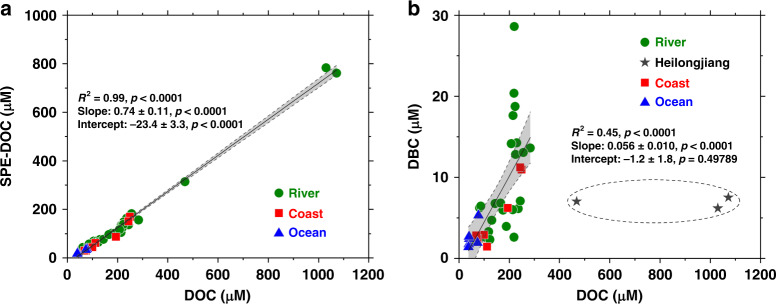
Correlations of SPE-DOC and DBC with DOC. **a** Concentrations of SPE-DOC vs. DOC for all samples determined (*n* = 40, *R*^2^ = 0.99, *p* < 0.0001); and **b** concentrations of DBC vs. DOC for the samples (*n* = 37, *R*^2^ = 0.45, *p* < 0.0001, Heilongjiang data were not included) (DOC dissolved organic carbon, SPE-DOC solid phase extracted dissolved organic carbon, DBC dissolved black carbon). The statistics are for the regression of the average values. The gray areas show the 95% confidence interval of the linear regression. We applied one-sided *F* test and two-sided *t* test, and the calculated *p* values as shown.

For the samples we studied, we found a very good linear correlation (*R*^2^ = 0.99, *p* < 0.001) between the concentrations of SPE-DOC with DOC in the river, coastal and ocean waters (Fig. 3a). This strong linear relationship could suggest an interesting phenomenon that, regardless the differences in DOC concentrations and the sample locations, the SPE method using the PPL cartridges likely extracted the same organic materials that were proportionally dissolved in DOC in natural waters. This fraction of SPE-DOC could have similar chemical properties such as molecular weight, composition and polarity^9,24,26^. In contrast, the DBC concentrations isolated in SPE-DOC also had a linear but relatively weak correlation (*R*^2^ = 0.45, *p* < 0.001) with DOC in the samples we studied (except for the Heilongjiang River). We think that this could be reasonable because the content of DBC, unlike the SPE-DOC fraction, is source-specific and should be largely controlled by the input of DBC source, while DOC could be derived from multiple sources such soil OC, biomass degradation and in situ production. The Heilongjiang River, for example, had the highest DOC concentrations (857 ± 337 μM) but much lower DBC contents (0.9 ± 0.5%) compared to other rivers. This could reflect relatively lower input of DBC in the less populated and cold northern climate with fewer landscape fires than the populated warm southern region with more biomass burning^13^. The lack of correlation between DBC and DOC concentrations has also been reported recently for the Amazon River and attributed to the divergent effects of soil properties, temperature, rainfall, and aerosol deposition on DOC and DBC mobilization from the catchments of tropical rivers^9^. Despite the different DBC isolation methods used, however, the DBC contents reported in the different rivers are in the comparable range of 4–10% in general. For example, based on the BPCA method, DBC has been reported to account for 7.0–9.9% of riverine DOC in the Paraíba do Sul^25^ and Amazon^9^ rivers, 7.3% in the Mississippi River and 6.9–8.4% in the Congo River^24^, comparable to the values of 3.7–7.6% for the six rivers we determined using the CTO method. For the four large continental rivers in China that we studied, based on the average discharge rates, measured DOC concentration and DBC/DOC ratio of each river, we calculated an annual flux of DBC of 5.5 × 10^10^, 3.5 × 10^9^, 2.2 × 10^10^, and 1.1 × 10^10^ g carried by the Yangtze, Yellow, Pearl, and Heilongjiang rivers, respectively. Together, 9.2 × 10^10^ g of DBC was transported by these rivers. This flux accounts for only a very small fraction (0.34%) of the 27 Tg of global riverine DBC export estimated by^12^ and is only 3.4–4.8% of the annual DBC flux (1.9–2.7 Tg) estimated for the Amazon River, the largest river in the world, largely due to the high DOC and DBC concentrations in the river^9^.

The variable DBC contents we determined in the rivers could also reflect the seasonal variations because we collected our samples during different seasons and years. This could be demonstrated by the monthly samples of the Yellow River. In general, lower DBC/DOC rations were measured in the winter months (January, November, and December) and summer months (June and July), with more rain precipitation and a relatively high flow rate^26^. This could suggest that the cold and frozen temperature in the winter in the lower Yellow River drainage region can reduce the release and dissolution of DBC from charcoal preserved in soils. On the other hand, more precipitation could dilute the DBC concentrations in the river. For most of the year, the DBC content was within the range of the other world rivers^9,24,25^.

For DBC in the ocean, a relatively large range of DBC/DOC has been reported in different studies. Using the BPCA method, Wagner et al.^24^ reported that very low DBC abundance accounted for 0.8–1.8% of the DOC in the Pacific and Atlantic oceans, while using the same method, Coppola and Druffel^10^ and Lewis et al.^27^ reported relatively higher DBC contents, accounting for 4.2–8.6% of the DOC in the Atlantic, Pacific, and Arctic oceans, which are comparable to the values we obtained in the ECS (2.9 ± 1.2%) and the Mariana Trench site in the western NP (2.6–6.8%).

**Fig. 4:**
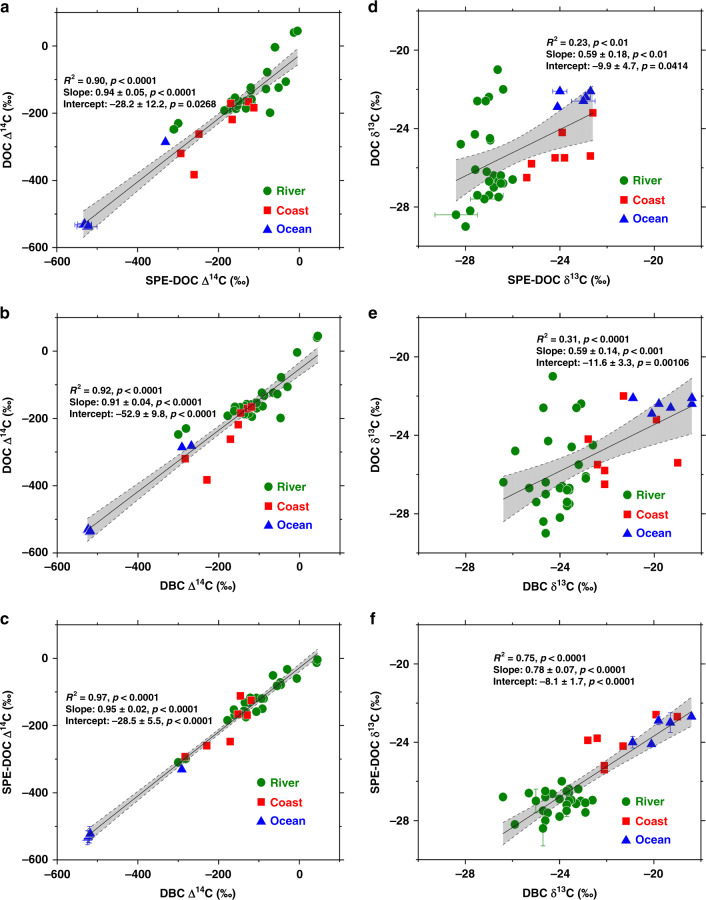
Isotopic correlations between different carbon pools. **a** DOC Δ^14^C vs. SPE-DOC Δ^14^C (*n* = 38), **b** DOC Δ^14^C vs. DBC Δ^14^C (*n* = 40), **c** SPE-DOC Δ^14^C vs. DBC Δ^14^C (*n* = 38); and **d** DOC δ^13^C vs. SPE-DOC δ^13^C (*n* = 39), **e** DOC δ^13^C vs. DBC δ^13^C (*n* = 40), and **f** SPE-DOC δ^13^C vs. DBC δ^13^C (*n* = 39) for the river, coastal and ocean samples (DOC dissolved organic carbon, SPE-DOC solid phase extracted dissolved organic carbon, DBC dissolved black carbon). The points with error bars are the average values of duplicate measurements and the error bars represent the range of the measured values. The statistics are for the regression of all data points. The gray areas show the 95% confidence interval of the linear regression. We applied one-sided *F* test and two-sided *t* test, and the calculated *p* values as shown.

The isotopic signatures of DOC, SPE-DOC, and DBC we studied for the various samples provide more insight for the transport and cycling of these different OC materials in rivers and oceans. As shown in Fig. 4, we see strong linear correlations for the plots of Δ^14^C values among DOC, SPE-DOC, and DBC. Good positive correlations exist between the Δ^14^C values of DOC vs. SPE-DOC (Fig. 4a, *R*^2^ = 0.90, *p* < 0.0001), DOC vs. DBC (Fig. 4b, *R*^2^ = 0.92, *p* < 0.0001), and SPE-DOC vs. DBC (Fig. 4c, *R*^2^ = 0.97, *p* < 0.0001). These strong correlations suggest that DOC, SPE-DOC, and DBC have systematic covariances in terms of their Δ^14^C values, and DBC was coupled with DOC and aged on the same time scales during transport from river to coast and open oceans. In the four rivers we studied, the ^14^C ages of riverine DBC are all younger (modern to 1510 years, except in the two small mountainous rivers in Taiwan) than the ^14^C ages of DOC (modern to 1720) especially in the Yangtze and Pearl rivers, consistent with the results reported for the Amazon River^9^. The relatively younger ^14^C ages of DBC in the rivers suggest that riverine DBC is mainly sourced from biomass burning rather than fossil fuel combustion. In our previous study, we calculated based on a two end-member isotope mass balance model that 78–85% of the DBC transported in the Yangtze and Yellow rivers was derived from biomass burning^26^. Regardless of the monthly variations of DBC contents in the Yellow River, their ^14^C ages (1143 ± 180) had less variation, supporting the source-specific input of DBC in the river. The same calculation reveals that 83 and 100% of the riverine DBC transported in the Pearl and Heilongjiang rivers and 65% of DBC in the Taiwan mountainous rivers are derived from biomass burning. The DBC was dissolved over time from the charcoal preserved in soils and entered waters in streams and rivers^12,25,28^. The older DBC in the Taiwan mountain rivers could suggest that some charcoals were preserved in soil for longer amounts of time. In contrast, the riverine DBC ^14^C ages are distinctively different than the PBC transported in rivers. Our previous study showed that PBC transported in the Yangtze and Yellow rivers had ^14^C ages of 4550 and 5830 years, respectively, as derived mainly from fossil fuel combustion^26^. For 18 global rivers, it was reported that the average PBC ^14^C age was 3700 ± 400 years, much older than the ^14^C age of DBC in the rivers^6^.

Very few ^14^C measurements of oceanic DBC have been reported in the literature. Ziolkowski and Druffel^11^ reported the first ^14^C measurement of DBC isolated by ultrafiltration in the Atlantic and Pacific oceans. The ^14^C ages of DBC were 15,680–18,300 years old in the Atlantic and 17,000–20,100 years old in the Pacific, much older than the bulk DOC ^14^C ages of 4000–6000 years in the 2 oceans^29,30^. More recently, using SPE-DOC isolation, Coppola and Druffel^10^ reported that the ^14^C ages of DBC were 4800 ± 620 years in the surface water and 23,000 ± 3000 years in the deep water of the Arctic, Atlantic, and Pacific oceans. They concluded that DBC in the ocean is not homogeneous; one younger DBC pool is cycling on centennial time scales, and one ancient DBC pool is cycling on >10^5^-year time scales^10^. The isotopic signatures of DBC in the ocean determined in our study, however, are sometimes inconsistent with these previous findings. For DBC in the oceans, we found that the average ^14^C ages were 1835 ± 616 years in the ECS; 2570 ± 184 years in the surface water; and 5900 ± 14 years and 5820 ± 28 years in the deep waters (3000–6000 m) and hadal depth (8000–10,000 m) at the Mariana Trench site in the NP, respectively. The DBC ^14^C ages were all younger than the bulk DOC ^14^C ages in the Yangtze River and Yellow River estuaries, and the ECS, and approximately the same as the DOC ^14^C ages in the surface water and slightly younger in the deep waters at the Mariana Trench site. Based on these results, we speculate that DBC in the ocean was transported and aged during its cycling through rivers and estuaries to coastal and open oceans in a continuum process coupled with DOC. DBC is likely cycled and aged on the same time scales as the bulk DOC pool in the ocean. Meanwhile, we remain puzzled about the DBC age differences found between our study and previous studies. It is possible that the age difference could be due to the CTO and BPCA methods used, which could have determined different organic compounds with very different ^14^C signatures. The relatively consistent Δ^14^C values of DBC we determined for the monthly samples in the Yellow River did suggest that we isolated the same materials. This age inconsistency is certainly an important question that needs to be further investigated.

Unlike their Δ^14^C signatures, the correlations between the δ^13^C values of DOC and SPE-DOC (Fig. 4d, *R*^2^ = 0.23, *p* < 0.01), DOC and DBC (Fig. 4e, *R*^2^ = 0.31, *p* < 0.001) are weak. In comparison, the correlation between SPE-DOC and DBC δ^13^C values is much better (Fig. 4, *R*^2^ = 0.75, *p* < 0.0001). This was due to the variable ranges of DOC δ^13^C values (−21.0 to −29.0‰) in the rivers we studied. Since δ^13^C in DOC is mainly an OC source indicator, the variable DOC δ^13^C values in the rivers indicate that DOC was derived from different sources. For DOC in the Heilongjiang River, for example, it was mainly derived from the leaching and degradation of plant biomass with modern ^14^C ages. Its DOC δ^13^C values (−28.2 to −29.0‰) were consistent and significantly depleted relative to the DOC δ^13^C values in the three large continental rivers, Yangtze, Yellow, and Pearl, where the sources of DOC were more complicated, including both natural and anthropogenic inputs^31,32^. As discussed above, the large variations of DOC δ^13^C values (−21.0 to −27.0‰) measured in the Yellow River could reflect seasonal source variations of DOC, consistent with the weak correlation between DBC and DOC concentrations (Fig. 3b). In contrast to bulk DOC, the good correlations between SPE-DOC and DBC δ^13^C values (Fig. 4f) again suggests that these organic fractions are more source-specific than other organic fractions.

**Fig. 5:**
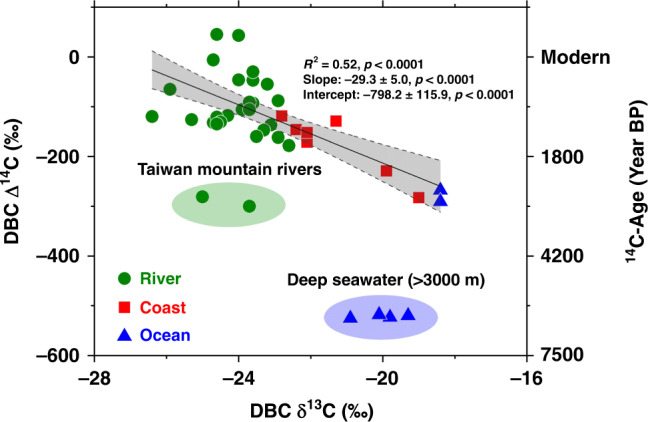
Integrated isotopic correlation of DBC. Plot of Δ ^14^C vs. δ^13^C values of DBC (dissolved black carbon) measured in the four rivers (Yangtze, Yellow, Pearl, and Heilongjiang), the Yangtze and Yellow river estuaries, the East China Sea (ECS) and the Mariana Trench site in the North Pacific. The statistics are for the regression of the average values (*n* = 34, the Taiwan mountain river and deep seawater data not included). We applied one-sided *F* test and two-sided *t* test and the gray area shows the 95% confidence interval of the linear regression with *p* values as shown.

In our study, we also found that the DBC δ^13^C values are all enriched (by 2–4‰ in general) relative to the δ^13^C values of bulk DOC and SPE-DOC in rivers, coastal waters and the open ocean (Fig. 1c). We examined the correlations between the ^14^C ages and δ^13^C values of DBC for the samples (Fig. 5). A linear correlation (*R*^2^ = 0.52, *p* < 0.0001) appears between DBC ^14^C ages and its δ^13^C values (except for the Taiwan river and deep ocean). When the DBC ages increase from the rivers to the ocean, its δ^13^C values become enriched. Riverine DBC has more depleted δ^13^C values than oceanic DBC. This phenomenon is consistent with the results reported recently by Wagner et al.^24^, who found that oceanic DBC was approximately 6–8‰ more enriched in δ^13^C than riverine DBC. Based on the δ^13^C differences, they concluded that oceanic DBC was not derived from rivers, so the DBC in rivers and oceans originates from different sources. As discussed above for the ^14^C ages, we believe that DBC in the ocean is likely transported mainly by rivers through estuaries and coasts into the open ocean as a continuum, considering that the major pathways mobilizing and transporting DBC (27 Pg per year) from the land to the ocean are rivers^12,25,27,28^, compared to the rather smaller source of DBC (0.002–0.006 Gt per year) derived from atmospheric deposition in the Northern Hemisphere^33^. Clearly, what happens to the riverine DBC in the ocean and the causes of the different DBC δ^13^C in rivers and ocean remain interesting questions, which need to be further studied.

Our long-team charcoal leaching experiments clearly demonstrated two important processes: (1) DBC was leached out from charcoal and dissolved in DOC with time, and (2) the released DBC was degraded by bacteria (Fig. 2). If we assume that 322 μM (as measured in the bacteria-inhibited case) was also leached out from the charcoal in the bacteria-active case, then this amount of DBC was completely degraded by bacteria. The measured DOC Δ^14^C values provided good evidence that no modern DBC was added to the DOC (−170‰) at the end of the experiment based on the initial DOC Δ^14^C value (−166‰). In contrast to the bacteria-active case, when bacterial activities were inhibited, the DOC Δ^14^C value (−90‰) was significantly increased at the end of the experiment. Based on the ^14^C mass balance and assuming that the DBC released from the wood charcoal had a modern Δ^14^C value of 20‰, 41% of the modern DBC was added to the DOC pool at the end of the bacteria-inhibited experiment, which was somehow lower than the 48% calculated from the increased DOC concentration (440–228 μM). This discrepancy could be because at the end of the bacteria-inhibited experiment, we also measured some bacteria (2.8 × 10^4^ cell mL^−1^) present in the water that consumed some DBC. For DOC δ^13^C, the value in the bacteria-active water (−25.1‰) was close to the original value (−24.6‰) but lower (−27.8‰) in the bacteria-inhibited water, which was close to the δ^13^C value of the locust tree wood charcoal (−27.0‰) used. These findings support the Δ^14^C results, indicating that DBC was leached from the charcoal and dissolved in DOC in the bacteria-inhibited case.

Our results provide direct evidence supporting the speculation that a large fraction of riverine DBC could be degraded in the estuaries and ocean^9,24^. Even though the dissolution of DBC is a slow process that is likely dynamically controlled by its water-solubility, this process could be significant in the natural environment because C-rich charcoal, a common residue of incomplete biomass combustion, is widely preserved in soils^34,35^, and a large fraction of this charcoal could be removed as DBC with time and transported by streams and rivers to the ocean^28,36–39^. The dominant DBC transported by the rivers is derived from biomass burning, and the DBC is labile and biodegradable. This could be the reason why we did not measure elevated DBC contents in the oceans. Our study suggests that DBC is unlikely to be a significant refractory DOC pool cycling in rivers and oceans, and DBC is likely aged on the same time scales as DOC cycling in the ocean.

## Methods

### Study sites and sample collection

**Fig. 6:**
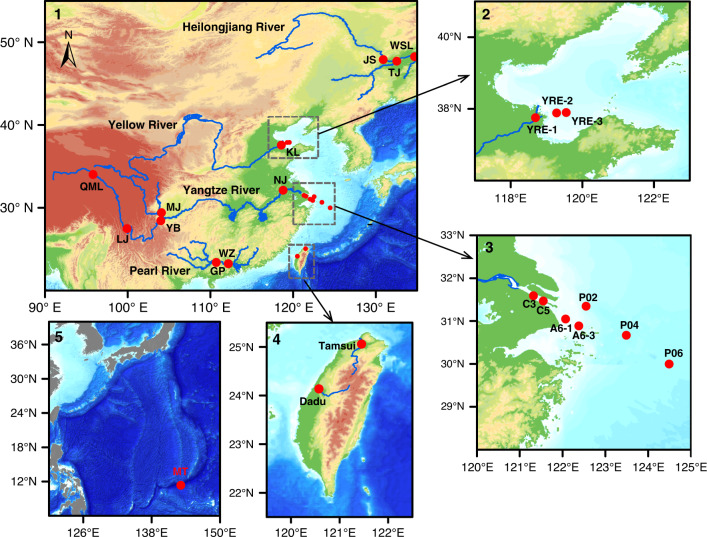
Map of the study sites and sampling locations. Sampling sites (1) in the four major continental rivers (Pearl, Yangtze, Yellow, and Heilongjiang) in China; (2) the three sites in the Yellow River Estuary; (3) the seven sites in the Yangtze River Estuary and the East China Sea (ECS); (4) the two sites in the two mountainous rivers in Taiwan; and (5) the Mariana Trench (MT) site (11°20.605′N, 142°19.557′E) in the western North Pacific Ocean.

Water samples used for this study were collected from four large rivers, namely, the Yangtze, Yellow, Pearl, and Heilongjiang rivers in China from 2015 to 2018 and from two small mountainous rivers (Dadu and Tamsui) in Taiwan in 2018. Samples were also collected from the Yangtze River and Yellow River estuaries, the ECS and the Mariana Trench site in the western North Pacific Ocean (NP) (Fig. 6). The detailed sampling locations and times are provided in Supplementary Table 1.

For the four rivers, in terms of the flow lengths, the Yangtze (6300 km), Yellow (5464 km), Heilongjiang (4400 km), and Pearl (2300 km) rivers rank as the four largest rivers in China, and the Yangtze River also ranks as the third longest river in the world. The drainage basins of these four rivers cover very different geographic environments from the southern subtropical warm climate to the far northern cold climate. The four rivers together drain 5.0 × 10^6^ km^2^ of China’s continental land and discharge ~1688 km^3^ yr^−1^ fresh water into the coastal seas. The two Taiwan mountainous rivers, Dadu and Tamsui, are relatively small (~200 km). In total, 13 sites were sampled along the rivers: 5 from the Yangtze River, 2 from the Pearl River, 1 from the Yellow River, 3 from the Heilongjiang River, and 1 each from the Dadu and Tamsui rivers. The Yellow River site was sampled monthly for a year in 2015^26,40^ and sampled again in June 2020. In addition to river sampling, seven samples (C3, C5, A6-1, A6-3, P02, P04, and P06) were collected from the Yangtze River Estuary and the ECS using an in situ pump (SFP-900P) on board the R/V Dongfanghong 2 in 2015^26,40^, and three samples (YRE-1, YRE-2, and YRE-3) were collected from the Yellow River and estuary on board a small R/V Xinda-18 on June 2, 2020 (Fig. 6). For the Mariana Trench site (11°20.605′N, 142°19.557′E; 10,800 m) in the NP, water samples were collected at six depths (50, 100, 3000, 6000, 8000, and 10,000 m) aboard R/V Dongfanghong 2 during a February–March 2017 cruise^41^.

At each site along the rivers and estuaries, 10–20 L of river water was collected using a precleaned stainless-steel bucket (washed with hot soapy water and rinsed with acid and Milli-Q water) in the main channel using a small boat (except for the Yellow River site where water was collected from a floating bridge, and the two small mountainous rivers in Taiwan where samples were collected by wading into the water). After collection, water was filtered through precombusted (550 °C for 4 h) 0.7 μm pore size GF/F filters and acidified to pH = 2 with high-purity HCl. Seawater samples were collected using 12 L Niskin bottles attached to a rosette sampler with conductivity, temperature, and depth sensors. Approximately, 30 L was collected from the Yangtze River Estuary and the ECS sites, and 50 L was collected from each depth at the Mariana Trench site. After collection, seawater was also filtered immediately and acidified to pH = 2. The acidified water samples were stored in precleaned polyethylene bottles at low temperature for further processing.

### SPE-DOC extraction

In the laboratory, acidified river and ocean water samples were extracted for DOC using the SPE method^42^. We used prepacked 60-mL volume PPL cartridges (Agilent Technologies Mega Bond Elute) that contained 5 g styrene divinyl benzene polymer as the sorbent (pore size 150 Å) with the capacity to process a large volume of water samples^40^. Before extraction, the cartridge was well cleaned according to the manufacturer’s guidelines. It was first rinsed with two bed volumes of high-purity methanol (B&J), followed by at least 2000 mL Milli-Q pure water to clean the sorbent free of OC (measured for DOC)^40^. Water samples were placed in a 2-L glass funnel, dropped directly into the top of the cleaned PPL cartridge, and passed through at a flow rate of 5 mL/min^40^. The permeate passing through the cartridge was frequently collected for DOC analyses to determine the extraction efficiencies (calculated based on the DOC concentration difference between water sample and permeate). Overall, there were average values of 61 ± 6% for riverine DOC, 51 ± 11% for estuarine and coastal DOC and 47 ± 4% for ocean DOC (Supplementary Table 2).

After extraction, the cartridge sorbent was rinsed with one bed volume of 0.01 M HCl twice to remove salts and then dried with high-purity nitrogen gas. Once the cartridge was dry, 80 mL high-purity methanol was used to elute the DOC extracted from the sorbent. We determined that >98% of DOC absorbed on the sorbent was eluted in the methanol phase^40^. The extracted DOC in methanol was then condensed to 5 mL using a Buchi Multivapor P-12 Evacuator. The condensed DOC was defined as SPE-DOC and stored in a refrigerator for further DBC and isotope determination. All glassware used in sample processing and storage was soaked in 10% HCl acid for 24 h, rinsed with Milli-Q water and baked at 550 °C for 5 h to remove any OC prior to use. Blanks associated with SPE extraction were determined. We considered two types of blanks during the extraction: a sorbent blank and a methanol elution blank. To test the blanks from the sorbent, we continuously collected Milli-Q water permeate for every 500 mL that passed through the precleaned cartridge for the same extraction volume (15 L) to measure DOC concentrations. To check the blanks during methanol elution, we collected 80 mL of methanol eluent, dried it with high-purity N_2_ gas, redissolved it in high-purity Milli-Q water, and measured its DOC concentration. In both cases, we found no detectible DOC (below the detection limit of ~3 μM) associated with either the sorbent or methanol^40^, consistent with the results reported in previous studies^42,43^.

### DBC isolation and measurement

The concentrations of DBC in the SPE-DOC were determined by the thermal oxidation method^21,44^. For DBC analysis, 1.0–2.0 mL of condensed SPE-DOC was added to a 9-mm outer diameter (OD) × 200-mm quartz tube (prebaked at 850 °C for 2 h) and dried with high-purity N_2_. The quartz tube was then placed in an oven, and the SPE-DOC was thermally oxidized at 375 °C for 24 h with a continuous air supply to remove non-BC OC. The OC left after thermal oxidation was defined as DBC. Following thermal oxidation, DBC was oxidized again in evacuated, flame-sealed quartz tubes (with CuO and Ag wire added) at 850 °C for 2 h in a muffle furnace. The resultant CO_2_ from combustion was collected cryogenically and quantitatively measured on a vacuum line. The amounts of DBC were calculated based on the volume of the resultant CO_2_.

To test the possibility that some non-BC DOC could be converted to DBC during thermal oxidation, we used three concentrated (10–15 mM) DOC samples: DOC leached from fresh salt marsh plants *Phragmites australis* and *Suaeda salsa* collected in the Yellow River Delta^45^ and DOC released from coastal phytoplankton collected in the ECS. One milliliter of each DOC was acidified and dried in a 9-mm OD × 200-mm quartz tube in triplicate and thermally oxidized at 375 °C for 24 h, the same as for the DBC treatment as described above. For all three DOC samples, we did not detect any measurable DBC converted from fresh DOC by the thermal oxidation method.

### DOC analysis

The concentration of DOC was analyzed by the high-temperature catalytic oxidation method using a Shimadzu TOC-L analyzer equipped with an ASI-L autosampler. The instrument was calibrated using 5-point calibration curves derived from a carbon standard solution of potassium hydrogen phthalate, and DOC values were checked against low carbon water and deep seawater reference materials (CRM, University of Miami, Rosenstiel School of Marine and Atmospheric Sciences). Blank subtraction was carried out using high-purity Milli-Q water that was analyzed before every five samples. The average blanks associated with DOC measurements were approximately 4 μM, and the analytic precision on triplicate injections was <3%^31^.

### Carbon isotope analyses

Carbon isotopes (Δ^14^C and δ^13^C) were analyzed for DOC, SPE-DOC, and DBC. SPE-DOC and DBC were dried and combusted in evacuated 9-mm OD quartz tubes, and the resultant CO_2_ was collected cryogenically on a vacuum line as described above. The purified CO_2_ was flame-sealed in a 6-mm OD glass tube for isotope analysis. The bulk DOC of the samples was processed separately using a modified UV oxidation method for carbon isotope measurement^46^. Briefly, approximately 200 mL (river) or 400 mL (seawater) of acidified water was placed into 2–4 custom-made 100-mL quartz reaction tubes specially designed to interface directly with a vacuum extraction line. Samples were first purged with ultrahigh purity (UHP) helium gas for 30 min to remove dissolved inorganic carbon and then irradiated using a 1.2 kW medium-pressure mercury arc UV lamp (Hanovia Co.) for 5 h. Following UV irradiation, CO_2_ generated from UV oxidation of DOC was purged again with UHP helium gas through the vacuum line and collected cryogenically and measured manometrically. The purified CO_2_ was flame-sealed in 6-mm OD Pyrex tubes for δ^13^C and Δ^14^C analyses. The oxidation efficiency and blanks associated with the UV oxidation of DOC were tested using high-purity Milli-Q water and a DOC (oxalic acid OXI) standard solution, yielding high oxidation efficiency (~95%) for DOC and considerably low blanks (<4 μg C)^46^.

The δ^13^C and Δ^14^C measurements were performed at the National Ocean Sciences Accelerator Mass Spectrometry (NOSAMS) facility at the Woods Hole Oceanographic Institution (WHOI) in the USA and the Center for Isotope Geochemistry and Geochronology (CIGG) of the Qingdao National Laboratory for Marine Science and Technology (QNLM) in Qingdao, China. Sample δ^13^C was analyzed using a Thermo 253-Plus isotope ratio mass spectrometer (IRMS) with a dual inlet at CIGG. The data reduction of δ^13^C values was performed using International Atomic Energy Agency (IAEA) isotope standards (IAEA-CH-3 Cellulose and IAEA-600 Caffeine), measured in ‰ with analytic precision of ≤0.2‰. Purified CO_2_ samples (>100 μg C) were first graphitized using a sealed tube zinc reduction method^47^, and ^14^C was analyzed using AMS. The Δ^14^C results were reported as fraction modern, and the conventional radiocarbon ages (YBP) were calculated based on Stuiver and Polach^48^. All Δ^14^C results were corrected for δ^13^C fractionation and for ^14^C decay for the time period between 1950 and the year of sample collection.

### DBC dissolution and degradation experiment

To test the dynamics of dissolution and biodegradation of DBC in rivers, we conducted long-term leaching experiments using freshly burned locust tree wood charcoal. The charcoal was obtained from a countryside family who used locust tree wood for cooking (see the charcoal image in Supplementary Fig. 1). We added 200 mg of charcoal (ground to 100–200 μm size) each into two 1-l glass bottles filled with filtered Yellow River water (collected in October 2015). In one bottle, 2.0 mL of saturated HgCl_2_ solution was added to eliminate bacterial activity (bacteria inhibited) and to compare the charcoal leaching process with the bacteria-active bottle. The bottles were covered loosely and incubated undisturbed at room temperature in the dark. During the incubation, the ground charcoal all deposited on the bottom, we left the bottles disturbed and no air or oxygen was bubbled into the water during the entire incubation period. Subsamples (10 mL) were collected from each bottle using a glass syringe at different times and were filtered with a precleaned 0.45 μm cellulose acetate membrane attached to a syringe for DOC concentration measurement. We assume that all DBC leached from the charcoal was dissolved in the water in the form of DOC. The leaching experiments were started in late October 2015, and after approximately 1000 days (June 30, 2018), the bottles were capped and kept at room temperature for another 700 days (May 10, 2020). We filtered the water and measured the final concentrations of DOC and DOC Δ^14^C and the δ^13^C values as described above. We also measured the bacterial abundance in the waters.

The bacterial abundance was measured in the charcoal leaching experiment waters. Filtered water samples (10 mL) were fixed with 2% paraformaldehyde (1:1 vol.) and stored at −80 °C before use. For bacterial abundance quantification, the samples were thawed at room temperature and then stained with 0.01% SYBR Green I (Invitrogen, CA, USA) at 37 °C for 1 h in the dark^49^. Yellowish green fluorescence beads (1 µm, Polysciences Inc., PA, USA) were added as an internal standard to calibrate and normalize the fluorescence and light scattering signals. Cell counting was conducted using a CytoFLEX flow cytometer (Beckman Coulter) equipped with a 488 nm argon laser (Supplementary Fig. 2). As a remedial measure, we measured the bacterial abundance in fresh Yellow River water collected at the same site on June 2, 2020 and assumed that the bacterial abundance was similar in the river water we used for the experiments.

## Supplementary information

Supplementary Information

## Data Availability

All data for this study are available in Supplementary Table 2 associated with the paper. The data will be publicly available on the Figshare data repository at 10.6084/m9.figshare.12774689.
