# Supplementary material for: Dissolved black carbon is not likely a significant refractory organic carbon pool in rivers and oceans
Source: Nat Commun. 2020 Oct 7;11:5051. doi: 10.1038/s41467-020-18808-8 (PMC7541478; doi:10.1038/s41467-020-18808-8)
Supplement: Supplementary file 1 — Supplementary Information [file 41467_2020_18808_MOESM1_ESM.pdf]

Supplementary Information

Dissolved black carbon is unlikely a significant refractory organic carbon pool in rivers and oceans

Yuanzhi Qi et al.

Supplementary Table 1. **Sampling information and site description**

Sample ID	Site	Sample Date	Location		Elevation	T	pH	Description
			°E	°N	m	(℃)		
Yangtze River								
QML	Qu Malai	2016.7.11	95.82	34.03	4191	14.2	8.6	Qinghai-Tibetan Plateau
LJ	Li Jiang	2016.10.14	100.08	27.01	2018	17.0	7.8	Hengduan Mountains
YB	Yi Bin	2016.10.15	104.65	28.76	321	17.0	7.5	Sichuan Basin
MJ	Min Jiang	2016.10.15	104.57	28.78	321	17.0	7.5	Sichuan Basin
NJ	Nan Jing	2016.3.29	118.79	32.13	10	14.0	7.7	Yangtze Plain, Lower reach
Yellow River								
January	Ken Li	2015.1.16	118.58	37.57	3	3.4	8.7	Agricultural Plains, lower reach
February	Ken Li	2015.2.14	118.58	37.57	3	3.2	8.5	Agricultural Plains, lower reach
March	Ken Li	2015.3.14	118.58	37.57	3	5.5	8.4	Agricultural Plains, lower reach
April	Ken Li	2015.4.17	118.58	37.57	3	16.2	8.4	Agricultural Plains, lower reach
May	Ken Li	2015.5.15	118.58	37.57	3	20.2	8.2	Agricultural Plains, lower reach
June	Ken Li	2015.6.12	118.58	37.57	3	26.5	8.3	Agricultural Plains, lower reach
July	Ken Li	2015.7.17	118.58	37.57	3	26.1	8.1	Agricultural Plains, lower reach
August	Ken Li	2015.8.14	118.58	37.57	3	29.0	8.6	Agricultural Plains, lower reach
September	Ken Li	2015.9.11	118.58	37.57	3	22.0	8.7	Agricultural Plains, lower reach
October	Ken Li	2015.10.16	118.58	37.57	3	17.0	8.6	Agricultural Plains, lower reach
November	Ken Li	2015.11.14	118.58	37.57	3	11.0	8.3	Agricultural Plains, lower reach
December	Ken Li	2015.12.11	118.58	37.57	3	4.5	8.1	Agricultural Plains, lower reach
Pearl								
GP	Gui Ping	2018.4.20	110.07	23.41	22	20.4	7.0	Hilly Regions, middle reach
WZ	Wan Zhou	2018.4.19	111.20	23.42	3	21.2	7.1	Hilly Regions, middle reach
Heilongjiang								
JS	Jin Sha	2017.9.15	130.90	47.91	65	13.9	7.0	Greater Khingan Mountains, middle reacl
TJ	Tong Jiang	2017.9.14	132.54	47.72	48	13.2	7.0	Sanjiang Plain, lower reach
WSL	Wusulijiang	2017.9.13	134.69	48.26	32	16.1	7.1	Sanjiang Plain, major tributary
Taiwan rivers								
Tamsui	Tamsui	2018.3.29	121.51	25.06	3	23.5	6.8	Taipei Basin, lower reach
Dadu	Dadu	2018.3.30	120.52	24.14	18	26.0	7.3	Western Taiwan Plain, lower reach
Yangtze River Estuary								
C3	S=0	2015.7.21	121.32	31.50		20.5		upper estuary
C5	S=0	2015.7.21	121.55	31.41		20.3		upper estuary
A6-1	YRE, S=14	2015.7.22	122.07	31.05		19.1		middle estuary
Yellow River Estuary								
YRE-1	S=0	2020.6.2	118.58	37.57		25.0	8.2	upper estuary
YRE-2	S=22	2020.6.2	119.53	37.42		21.0	8.0	middle estuary
YRE-3	S=28	2020.6.2	119.26	37.27		18.0	7.9	lower estuary
East China Sea (ECS)								
A6-3	ECS, S=19	2015.7.22	122.38	30.89		18.1		lower estuary
P02	ECS, S=28	2015.10.22	122.55	31.35		22.2		coastal region
P04	ECS, S=33	2015.10.28	123.49	30.67		23.9		coastal region
P06	ECS, S=34	2015.10.28	124.49	30.00		23.5		offshore
Mariana Trench, NP								
Water column	50 m	2017.2.26	142.91	11.36		28.6		Western North Pacific Ocean
	100 m	2017.2.23	142.91	11.36		26.1		Western North Pacific Ocean
	3000 m	2017.2.24	142.91	11.36		1.6		Western North Pacific Ocean
	6000 m	2017.2.26	142.91	11.36		1.4		Western North Pacific Ocean
	8000 m	2017.2.26	142.91	11.36		1.4		Western North Pacific Ocean
	10000 m	2017.2.27	142.91	11.36		1.4		Western North Pacific Ocean

Supplementary Table 2. Data summary for concentrations of DOC, DBC and isotope values measured for DOC, SPE-DOC and DBC in rivers and ocean (DOC: dissolved organic carbon; SPE-DOC: solid phase extracted DOC; DBC: dissolved black carbon)

**Study Area**						**DOC**			**SPE-DOC**			**DBC**		
		Salinity	DOC µM	SPE Efficiency %	DBC/DOC %	$\delta^{13}C$ ‰	$\Delta^{14}C$ ‰	Age Year BP	$\delta^{13}C$ ‰	$\Delta^{14}C$ ‰	Age Year BP	$\delta^{13}C$ ‰	$\Delta^{14}C$ ‰	Age Year BP
**Yangtze River**	QML	0	175	58	3.4	-26.6	-170	1490	-26.0	-118	950	-23.9	-106	835
	LJ	0	62	70	3.7	-26.8	-199	1720	-26.4	-72	535	-23.6	-47	320
	YB	0	105	66	2.5	-27.5	-106	840	-26.6	-33	210	-23.6	-30	180
	NJ	0	118	59	2.8	-24.8	-124	1000	-28.2	-51	350	-25.9	-65	475
	MJ	0	230	63	6.2	-26.7	-78	590	-26.9	-79	600	-24.0	-46	315
	Mean		138±65	63±5	3.7±1.5	-26.5±1.0	-135±49	1128±466	-26.8±0.8	-71±32	529±281	-24.2±1.0	-59±29	425±252
**Yellow River**	January	0	236	69	2.6	-26.4	-164	1380	-26.8	-128	1040	-26.4	-120	965
	February	0	225	66	5.7	-22.6	-188	1610	-27.5	-175	1480	-24.7	-132	1080
	March	0	215	63	8.2	-21.0	-195	1680	-26.6	n.d.	n.d.	-24.3	-118	945
	April	0	220	61	13.0	-26.7	-186	1600	-26.6	-132	1070	-25.3	-126	1020
	May	0	131	58	3.6	-27.0	-159	1330	-26.8	-118	940	-24.6	-121	970
	June	0	123	52	1.9	-24.3	-158	1320	-27.6	-162	1350	-24.5	-131	1070
	July	0	214	49	2.8	-26.4	-164	1380	-26.5	-132	1080	-24.6	-135	1100
	August	0	219	62	9.3	-22.4	-187	1600	-27.0	-156	1300	-23.1	-137	1120
	September	0	284	55	4.8	-22.6	-167	1410	-27.1	-166	1395	-23.3	-147	1230
	October	0	244	56	2.9	-24.6	-166	1390	-26.9	-169	1420	-23.5	-160	1340
	November	0	188	54	2.1	-24.5	-192	1650	-27.0	-185	1575	-22.6	-178	1510
	December	0	219	57	1.2	-26.1	-178	1520	-27.6	-153	1270	-22.9	-162	1360
	Mean		210±45	59±6	4.8±3.6	-24.6±2.0	-175±14	1489±134	-27.0±0.4	-152±22	1265±205	-24.2±1.1	-139±19	1143±180
**Pearl River**	GP	0	82	61	7.6	-26.2	-132	1065	-27.1	-120	960	-22.9	-88	675
	WZ	0	87	66	7.4	-26.7	-124	995	-27.0	-120	965	-23.6	-93	715
	Mean		85	64	7.5	-26.5	-128	1030	-27.1	-120	963	-23.3	-91	695
**Heilongjiang River**	JS	0	1030	76	0.6	-28.2	40	>Modern	-27.8	-13	35	-24.0	43	>Modern
	TJ	0	1072	71	0.7	-29.0	45	>Modern	-28.0	-4	>Modern	-24.6	45	>Modern
	WSL	0	468	67	1.5	-28.4	-4	>Modern	-28.4±0.9	-60±3	433±25	-24.7	-6	>Modern
	Mean		857±337	71±5	0.9±0.5	-28.5±0.4	27±27	>Modern	-28.1±0.3	-25±30	234±281	-24.4±0.4	27±29	>Modern
**Taiwan rivers**	Dadu	0	223	62	8.4	-27.4	-230	2035	-27.0±0.6	-299±2	2785±21	-25.0	-281	2590
	Tamsui	0	208	56	6.8	-27.6	-248	2220	-27.2±0.6	-310±10	2908±81	-23.7	-300	2800
	Mean		216	59	7.6	-27.5	-239	2128	-27.1	-305	2847	-24.4	-291	2695
**Yangtze River Estuary**	C3	0	144	53	4.7	-27.4	-164	1375	-27.5	-150	1235	-23.7	-91	705
	C5	0	166	58	4.1	-26.8	-154	1280	-26.5	-159	1320	-23.7	-106	830
	A6-1	14	194	45	3.2	-22.0	-171	1450	-24.2	-169	1420	-21.3	-129	1050
	Mean		168±25	52±7	4.0±0.8	-25.4±3.0	-163±9	1368±85	-26.1±1.7	-159±10	1325±93	-22.9±1.4	-109±19	862±175
**Yellow River Estuary**	YE-1	0	256	71	5.1	-25.5	-128	1035	-26.4	-82	620	-23.2	-55	390
	YE-2	22	249	68	4.4	-24.2	-166	1395	-23.9	-125	1000	-22.8	-119	950
	YE-3	28	244	62	4.6	-25.5	-184	1565	-23.8	-112	880	-22.4	-146	1200
	Mean		250±6	67±5	4.7±0.4	-25.1±0.8	-159±29	1332±271	-24.7±1.5	-106±22	833±194	-22.8±0.4	-107±47	847±415
**East China Sea (ECS)**	A6-3	19	112	55	1.3	-26.5	-262	2370	-25.4	-248	2230	-22.1	-171	1440
	P02	28	100	44	2.9	-25.8	-219	1920	-25.2	-166	1390	-22.1	-152	1260
	P04	33	74	40	3.3	-25.4	-320	3033	-22.7	-293	2720	-19.0	-283	2620
	P06	34	69	43	4.1	-23.2	-383	3817	-22.6	-260	2350	-19.9	-229	2020
	Mean		89±21	46±7	2.9±1.2	-25.2±1.4	-296±71	2785±826	-24.0±1.5	-242±54	2173±562	-20.8±1.6	-209±59	1835±616
**Mariana Trench-Upper depth**	50 m	34	78	47	6.8	-22.4	-282	2660		n.d.		-18.4	-268	2440
	100 m	34	74	45	2.6	-22.1	-286	2700	-22.7	-331	3160	-18.4	-291	2700
	Mean		76	46	4.7	-22.3	-284	2680	-22.7	-331	3160	-18.4	-280	2570
**Mariana Trench-Deep depth**	3000 m	34	39	47	4.5	-22.1	-532	6100	-24.0±0.3	-534±21	6140±106	-20.9	-525	5910
	6000 m	34	41	54	5.9	-22.4	-529	6050	-22.9±0.2	-531±17	6100±81	-19.8	-523	5890
	Mean		40	51	5.2	-22.3	-531	6075	-23.5	-533	6120	-20.4	-524	5900
**Mariana Trench-Hadal depth**	8000 m	34	38	45	3.7	-22.6	-538	6180	-23.0±0.5	-525±25	5920±147	-19.3	-520	5840
	10000 m	34	40	44	6.7	-22.9	-535	6150	-24.1	-522	5870	-20.1	-518	5800
	Mean		39	45	5.2	-22.8	-537	6165	-23.5	-524	5895	-19.7	-519	5820

Note: The mean error represents the standard deviation of the corresponding data in each study area, and the error for individual sample represents the range of the duplicate measurements. The Yellow River and ECS (P02, P04) data are from Wang et al. (2016)[14].

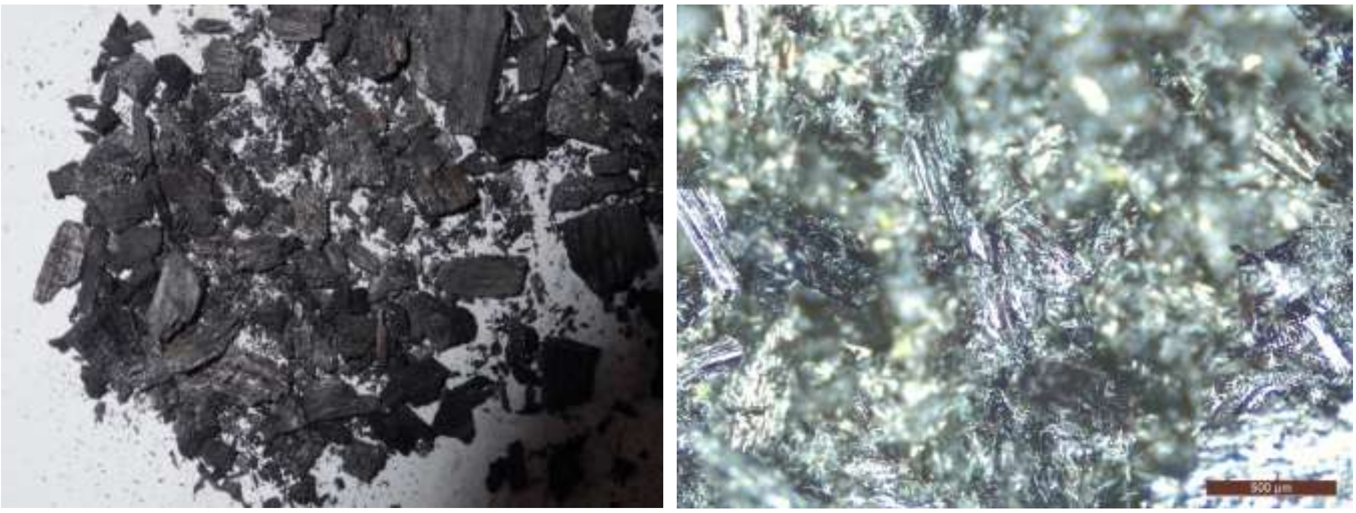

Supplementary Figure 1. **Photo of wood charcoals used for the leaching experiments.** (left) the charcoal pieces of burned locust tree wood and (right) microscope image of charcoal particles used for leaching experiments (scale 500 µm). The image was taken using a Leica S9i Stereo Microscope.

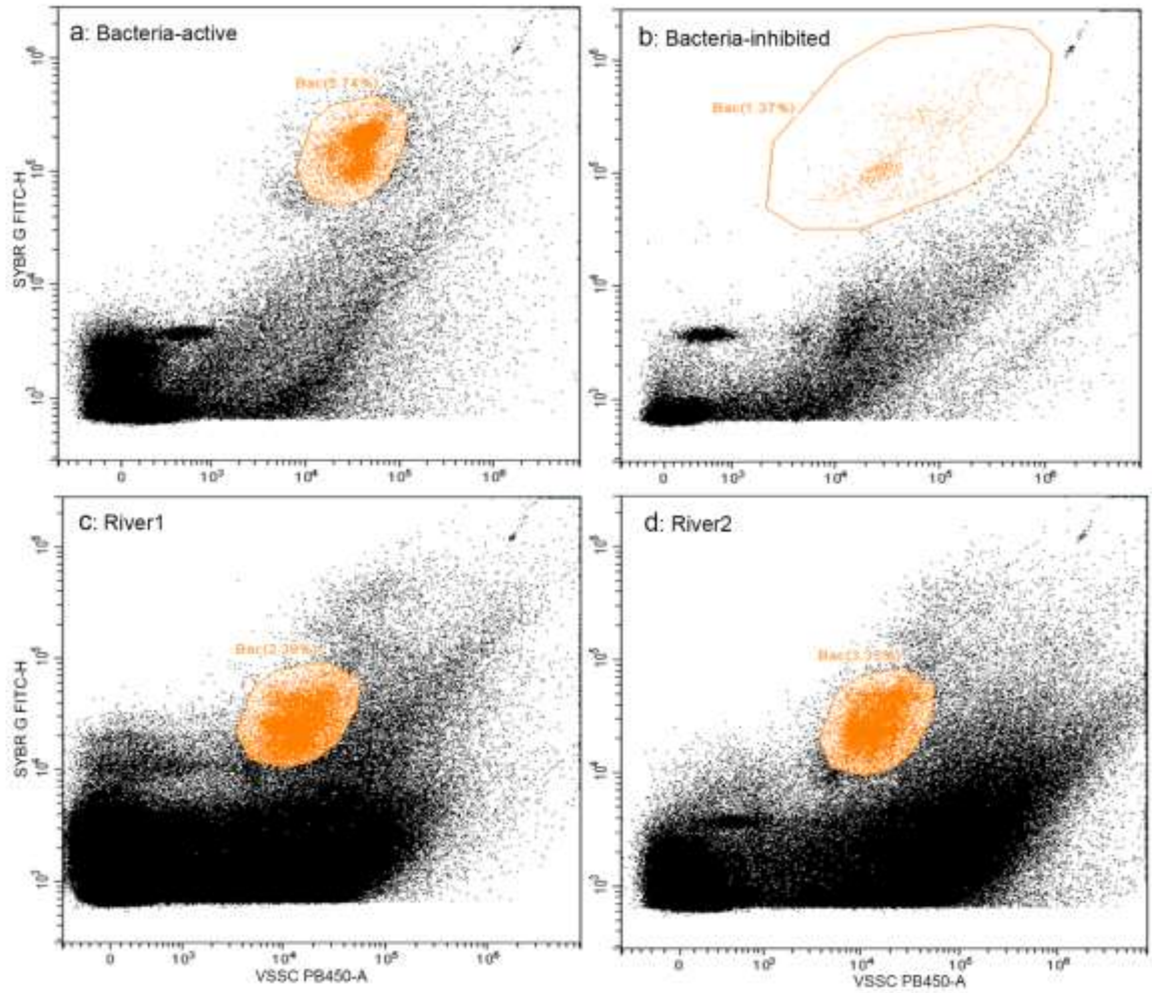

Supplementary Figure 2. **The bacterial abundance in waters.** The bacterial abundance was measured in (a) bacteria-active and (b) bacteria-inhibited waters at the end of the charcoal leaching experiments; and in (c, d) the filtered fresh Yellow River waters (duplicates) collected on June 2, 2020. The bacterial abundance of Gating strategies was determined using a CytoFLEX flow cytometry.
